# Relationship between hemodynamic parameters and portal venous pressure in cirrhosis patients with portal hypertension

**DOI:** 10.1515/biol-2020-0101

**Published:** 2020-12-31

**Authors:** Hongjuan Yao, Yongliang Wang

**Affiliations:** Department of Gastroenterology, Xi’an Gaoxin Hospital, Xi’an, 710075, China; Gerontological Surgery Department, Xi’an International Medical Center, Xi’an, 710100, China

**Keywords:** portal hypertension, hemodynamics, Doppler, portal venous pressure, statistical analysis

## Abstract

Cirrhosis caused by viral and alcoholic hepatitis is an essential cause of portal hypertension (PHT). The incidence of PHT complication is directly proportional to portal venous pressure (PVP), and the clinical research of PVP and its hemodynamic indexes is of great significance for deciding the treatment strategy of PHT. Various techniques are currently being developed to decrease portal pressure but hemodynamic side effects may occur. In this article, the hemodynamic indexes of cirrhotic PHT patients were studied to explore the correlation between the index and PVP and to evaluate the clinical value of Doppler ultrasound in measuring PVP in patients with PHT. This was achieved by selecting 90 cirrhotic PHT patients who underwent transjugular intrahepatic portosystemic shunt in our hospital from June 2015 to September 2019. Fifty healthy people who had a physical examination in the hospital in the same period were selected as the control group. The liver hemodynamic parameters of two groups were measured by Doppler ultrasound, and the cirrhotic PHT patients were graded by the Child–Pugh grading method to evaluate the liver function and measure the PVP value. The results showed that both the central portal vein velocity (PVV) and splenic vein velocity (SVV) of the PHT group were lower than those of the control group. Also, the portal vein diameter (PVD), portal venous flow and splenic vein diameter (SVD) were higher than those of the control group (all *P*s < 0.05). Among liver function graded PHT patients, the PVD, PVV, SVD and SVV were significantly different (all *P*s < 0.05). Furthermore, the PVP of patients with liver function grades A, B and C was 38.9 ± 1.4, 40.6 ± 5.1 and 42.5 ± 4.8 cmH_2_O, respectively, with a significant difference. It can be concluded from this study that Doppler ultrasound can be used as a tool for clinical assessment of PHT in cirrhosis patients. Doppler ultrasound showed a good prospect in noninvasive detection of PHT in cirrhosis; however, this technique needs application on large sample population study to validate the results.

## 1 Introduction

Portal hypertension (PHT) is one of the most common severe complications in patients with liver cirrhosis [1,2,3]. The detection of portal hemodynamic parameters is currently one of the effective means to evaluate the efficacy of drug therapy on PHT. Cirrhosis patients with severe impairment of liver function, mainly grade C patients who are at high risk, are often unable to tolerate general surgery. Therefore, it is imperative to assess the hemodynamics of the patients before surgery to adopt appropriate surgical methods to reduce bleeding and complications [4,5,6]. The standard reference value of portal venous pressure (PVP) is 6.7–13.3 cmH_2_O (i.e., 0.6566–1.3034 kPa); and when the PVP value exceeds 1.3034 kPa, PHT can be clinically diagnosed [6]. Cirrhosis due to viral and alcoholic hepatitis is an essential cause of PHT. Studies show that the incidence of PHT complication is directly proportional to PVP, and the clinical research of PVP and its hemodynamic indexes is of great significance for the treatment of PHT [7,8,9,10].

Various techniques are currently being developed to decrease the portal pressure but hemodynamic side effects may occur. Thus, a detailed study and characterization of portal venous, splanchnic and systemic hemodynamic parameters are needed to decide on the treatment of PHT and evaluate its efficacy [11]. Since these hemodynamic abnormalities progress with liver disease, they are essential for developing a treatment strategy. This study was designed to evaluate the relationship between hemodynamic parameters and PVP in cirrhotic PHT patients for clinical application in designing the treatment strategies. In this article, we used the ultrasonic Doppler measurement technology to measure the liver hemodynamic indexes of the patients including PVP, splenic blood flow, central portal vein velocity (PVV), splenic vein velocity (SVV), portal vein diameter (PVD), portal venous flow (PVF) and splenic vein diameter (SVD). The correlation between the relevant indexes and PVP was analyzed, and the reliability and effectiveness of the ultrasonic Doppler technology for the evaluation of PVP were discussed, so that the degree of PHT in cirrhosis can be assessed and the PVP monitored.

## 2 Patients and methods

### 2.1 Patients

Ninety cirrhotic PHT patients who underwent transjugular intrahepatic portosystemic shunt (TIPS) in our hospital during the period from June 2015 to September 2019 were selected as the study subjects. The diagnosis of clinical PHT was mainly based on physical signs, previous medical history, clinical symptoms, B-mode ultrasound, computed tomography, endoscopic examination, blood routine examination, viral hepatitis markers, etc. All patients were clinically diagnosed with PHT, and the PVPs of all patients were greater than 13.3 cmH_2_O. The Child–Pugh grading method was employed to evaluate the liver function of PHT patients. Fifty healthy people who had a physical examination in the hospital in the same period were selected as the control group.


**Informed consent:** Informed consent has been obtained from all individuals included in this study.
**Ethical approval:** The research related to human use has been complied with all the relevant national regulations, institutional policies and in accordance with the tenets of the Helsinki Declaration and has been approved by the authors’ institutional review board or equivalent committee (study approval number BFG201Y98).

### 2.2 Inclusion and exclusion criteria

The inclusion of patients was made based on the following criteria [12,13]: (1) age ≥ 18 years; (2) healthy liver and kidney functions, normal blood lipid profile, no history of liver and venereal diseases and negative hepatitis B and C virus results; (3) no contraindication of operation and narcotic medicines; (4) signing the informed consent of treatment and nursing methods voluntarily.

The exclusion of patients was made based on the following criteria [14,15]: (1) patients with TIPS treatment contraindications or unable to withstand TIPS treatment procedure; (2) patients with portal vein thrombosis or upper gastrointestinal bleeding symptoms within 14 days; (3) patients with a history of medications that affect hemodynamics, such as the use of calcium channel blockers, β-receptor blockers, angiotensin-converting enzyme inhibitors, diuretics, or with a history of operations affecting hemodynamics, such as liver surgery and esophageal ligation; (4) pregnant or lactating women; (5) patients having incomplete clinical data.

### 2.3 Measurement of liver hemodynamic indexes

Doppler ultrasound was used for index detection 1–2 days before TIPS. Before the screening, the subjects in both groups were required to abstain from food and water for more than 12 h. The probe frequency of Doppler ultrasound examination instrument was set at 3.5 MHz. After lying down for 5 min, the device began to take samples with a volume of about 2–6 mm. Abdominal blood flow mode was selected. The central PVV, SVV and PVF of the patients were measured. The PVF diameter (PVD) was calculated as PVF = 60 × π × (PVD/2)^2^ × PVV.

### 2.4 PPV measurement method

During TIPS, the tool entered the right branch of the hepatic vein through the right internal jugular vein and then punctured after adjustment. After the successful puncture of the portal vein, Cobra angiography catheter was inserted into the central portal vein along with the guide wire before balloon expansion, and the pressure was measured with a glass tube water column pressure gauge [16,17].

### 2.5 Statistical method

The data were processed and analyzed by using SPSS software (version 22.0; SPSS Inc., Chicago, USA). The metrological parameters were expressed as x̄ ± SD where x̄ is the mean and SD is the standard deviation, and *t* test was applied for two independent samples. Student-Newman-Keuls-*q* (SNK-*q*) test was adopted for statistical analysis of intragroup comparison, and one-way analysis of variance was used for intergroup comparison. The count data were expressed as % and *χ* ^2^ test was used for statistical analysis. *P* < 0.05 indicated that the difference was statistically significant. Variables such as PVV, PVF, PVD, SVD and SVV were compared between groups.

## 3 Results

### 3.1 Comparison of liver hemodynamic parameters between the two groups

Of 90 study subjects, 64 were males and 26 females. The age of the patients ranged from 28 to 63 years, with an average age of 50.7 ± 8.6 years. Most of the patients were hepatocirrhotic caused by hepatitis B accompanied by ascites and/or esophageal varices. All patients were clinically diagnosed with PHT, and the PVPs of all patients were greater than 13.3 cmH_2_O. The Child–Pugh grading method (Table 1) was employed to evaluate the liver function of the PHT patients. The liver function of PHT patients was classified as follows: 28 patients with grade A, 40 patients with grade B and 22 patients with grade C [18,19,20]. Fifty healthy people who had a physical examination in the hospital in the same period were selected as the control group, which comprised 30 males and 20 females, aged from 25 to 60 years, with an average age of 45.1 ± 6.6 years. No significant difference was observed in age and gender between two groups (both *P* > 0.05).

**Table 1:** Child–Pugh rating standard of liver function in cirrhotic patients

Index	Points		
	1	2	3
Encephalopathy	None	Levels 1–2	Levels 3–4
Ascites	None	Mild	Medium to severe
Serum bilirubin (µmol/L)	<34	34–51	>51
Albumin (g/L)	>35	28–35	<28
Increased prothrombin time (s)	<4	4–6	>6

Note: grade A: 5–6 points; grade B: 7–9 points; grade C: ≥10 points.

A comparison of the liver hemodynamic parameters of two groups (PHT group and control group) showed that the PVV and SVV values of the PHT group were significantly lower than those of the control group. In contrast, the PVD, PVF and SVD values of the PHT group were considerably higher than those of the control group (all *P*s < 0.05; Table 2).

**Table 2:** Comparison of liver hemodynamic parameters between the two groups

Group	Number of cases	PVD (mm)	PVV (cm/s)	PVF (mL/min)	SVD (mm)	SVV (cm/s)
PHT group	90	15.7 ± 1.7	10.0 ± 1.7	1301 ± 157	11.1 ± 1.9	11.3 ± 1.6
Control group	50	9.9 ± 1.0	16.1 ± 0.7	735 ± 115	6.5 ± 0.9	15.9 ± 1.1
T		4.938	4.667	4.822	4.007	3.595
P		<0.001	<0.001	<0.001	<0.001	<0.05

Note: PHT = portal hypertension; PVD = diameter of main vein pressure; PVF = blood flow of portal vein; PVV = velocity of portal vein blood flow; SVV = velocity of splenic vein blood flow; SVD = diameter of splenic vein.

### 3.2 Comparison of hemodynamic parameters in PHT patients with different liver function grades

According to the comparison results of hemodynamic parameters of patients with various liver function grades, no significant difference was observed in PVF of patients of all categories (*P* = 0.344 > 0.05). Still substantial differences were observed in PVD, SVD, PVV and SVV in patients with different liver function grades (all *P*s < 0.05; Table 3). It can be seen from Table 3 that the PVD and SVD values of patients with grades B and C were higher than those of patients with grade A, and the PVV and SVV values of patients with grades B and C were lower than those of the patients with grade A.

**Table 3:** Comparison of liver hemodynamic parameters among PHT patients with different liver function grades

Group	Number of cases	PVD (mm)	PVV (cm/s)	PVF (mL/min)	SVD (mm)	SVV (cm/s)
Grade A	28	14.6 ± 1.3	12.1 ± 1.2	1217 ± 138	10.1 ± 1.3	12.9 ± 1.4
Grade B	40	15.8 ± 1.3	8.8 ± 0.7	1277 ± 125	10.9 ± 1.5	10.9 ± 1.1
Grade C	22	17.7 ± 1.4	7.1 ± 0.6	1099 ± 154	12.5 ± 2.2	9.6 ± 0.8
F		23.445	87.881	1.115	4.931	38.622
P		<0.001	<0.001	0.344	<0.05	<0.001

Note: PVD = diameter of main vein pressure; PVF = blood flow of portal vein; PVV = velocity of portal vein blood flow; SVV = velocity of splenic vein blood flow; SVD = diameter of splenic vein.

### 3.3 Correlation between PVP and PVD in PHT patients with different liver function grades

The study of PVD in PHT patients with grade A, B and C liver function showed that the diameter increased with an increase in the liver function grade, but no significant difference was observed between the different grades. There was only a substantial correlation between PVP and PVD in PHT patients with grade A liver function, indicating that PVD is not a sensitive index to evaluate PVP (Table 4).

**Table 4:** Correlation between PVP and PVD in PHT patients with different liver function grades

Group	Number of cases	Pressure value (cmH_2_O, *x* ± *s*)	PVD	
			*r*	*P*
Grade A	28	38.9 ± 1.4	0.591	<0.05
Grade B	40	40.6 ± 5.1	0.385	>0.05
Grade C	22	42.5 ± 4.8	0.332	>0.05

### 3.4 Correlation between PVP and PVF in PHT patients with different liver function grades

The study of PVF in PHT patients with various liver function grades showed that PVF of grades A and B was higher than that of the control group. Still no significant difference was observed between grades A and B. Meanwhile, the difference between grades C and A or grade B was substantial (Table 5). By statistical analysis of linear regression equation, the correlation equation between PVP and PVF can be expressed as PVP = 1.8176 ± 0.0023 PVF. Therefore, it is very convenient to obtain PVP by control of color Doppler ultrasound in the clinic.

**Table 5:** Correlation between PVP and PVF in PHT patients with different liver function grades

Group	Number of cases	Pressure value (cmH_2_O, *x* ± *s*)	PVF	
			*r*	*P*
Grade A	28	38.9 ± 1.4	0.689	<0.01
Grade B	40	40.6 ± 5.1	0.597	<0.01
Grade C	22	42.5 ± 4.8	0.615	<0.05

### 3.5 Correlation between PVP and PVV in PHT patients with different liver function grades

The study of PVV in PHT patients with grade A, B and C liver function showed that with an increase in liver function grade, the PVV of patients with grade C was significantly lower than that of patients with grades A and B. Still no significant difference was observed between them (all *P*s > 0.05). The correlation between PVP and PVV was significant only in patients with grade A liver function (*P* < 0.05; Table 6). The dynamic detection of PVV has potential value in the diagnosis and liver function evaluation of cirrhotic PHT patients.

**Table 6:** Correlation between PVP and PVV in PHT patients with different liver function grades

Group	Number of cases	Pressure value (cmH_2_O, *x* ± *s*)	PVV	
			*r*	*P*
Grade A	28	38.9 ± 1.4	0.533	<0.05
Grade B	40	40.6 ± 5.1	0.322	>0.05
Grade C	22	42.5 ± 4.8	0.317	>0.05

## 4 Discussion

In most patients with liver cirrhosis, the portal and systemic hemodynamic changes occur. In liver cirrhosis, increased intrahepatic vascular resistance induces increased portal pressure and portosystemic shunt. These changes cause systemic hyperdynamic circulation, such as increased cardiac production and reduced systemic vascular resistance. The hyperdynamic systemic circulation and an improved splanchnic flow result in increased portal inflow and PHT maintenance [21].

The PVP value is affected by the resistance of the portal vein and PVF. The PVF has a major effect on liver regeneration, and reversible damage to hepatocytes begins immediately following graft recirculation [22]. When PVP keeps rising and exceeds 20 cmH_2_O clinically (the PVP of healthy people is between 6.7 and 13.3 cmH_2_O), it can be clinically diagnosed as PHT [23]. In cirrhotic PHT patients, PVP increases sharply, even to 50 cmH_2_O because of the increase in portal vein resistance and blood flow [24]. PVP index can be replaced by the hepatic venous pressure gradient (HVPG), and HVPG can be used as the best prognosis index for patients with cirrhosis [25]. In this context, given the importance of PHT in the natural history of cirrhosis patients, HVPG measurement will be expected to carry the prognostic details. Theoretically, the use of HVPG as a prognostic tool has many advantages: (1) it is an objective and continuous variable; (2) it improves in the presence of various clinical treatments and/or shows an improvement in the function of the liver; and (3) it has been extensively tested in cross-sectional, longitudinal, randomized controlled trials and meta-analysis to make it one of the most effective markers [26]. However, several disadvantages limit its use, namely, the measurement of HVPG is traumatic and prone to thrombosis, the costs associated with the methodology, the need of trained physicians to get a reliable measurement and the relative invasiveness of the methodology, all of which limit its application as a routine clinical test [27,28]. At present, Doppler ultrasound is often used to evaluate the PVP of patients with cirrhosis, and the noninvasive advantage of Doppler ultrasound makes it a routine method for clinical evaluation of PVP [29]. The parameters of liver hemodynamics, such as PVD and SVD, can be measured by Doppler ultrasound, which generates a foundation for noninvasive diagnosis of PVP.

Based on the comparison of liver hemodynamic parameters between the two groups, PVV and SVV in the PHT group were significantly lower than those in the control group, and PVD, PVF and SVD were markedly higher than those in the control group (all *P*s < 0.05). The results showed that the liver dynamics of PHT patients was in a highly circulatory state, the blood flow of the liver increased, the blood vessels expanded, the peripheral vascular resistance and arterial pressure decreased and the blood flow velocity decreased. The high dynamic, visceral circulation aggravated the increase in PVP. The clinical manifestations included increase in PVF and a significant decrease in vascular resistance of the visceral artery. The portal venous velocity and PVF considerably increased after the reperfusion of a liver graft and then returned to the baseline value, which was measured before hepatic parenchymal transection. The visceral hyperdynamic flow is not only an important reason for the continuous expansion of PVP but also an important factor for the occurrence of hepatorenal syndrome and visceral effusion [23]. In this study, significant differences were observed among PVD, SVD, PVV and SVV of patients with various liver function grades (all *P*s < 0.05). Previous study documented that the wider the PVD and SVD, the slower the blood flow of the portal vein system after operation and, therefore, the greater the possibility of thrombosis. Previous studies have found correlations between PVP or PVF and the degree of graft regeneration, which were measured at different postoperative time points. However, there is still no consensus about which portal venous hemodynamic parameter is of paramount importance in predicting the degree of graft regeneration [30,31,32].

PVD and SVD of patients increased with an increase in liver function grade, and only PVD of grade A PHT patients was correlated with PVP, indicating that PVD was not a sensitive index to evaluate PVP but only a reference index in clinical diagnosis. Generally, for patients with decompensated cirrhosis, PVD is proportional to the degree of PHT [33]. At the same time, the study shows that the clinical detection of PVD value will be affected by many factors, such as the patient’s position, body type, tester’s technical means, technical level and a professional degree. Besides, no significant relationship was observed between PVD increase and PHT in cirrhosis. PVD is not an accurate method to evaluate PVP clinically.

The comparative result of PVP and PVF in PHT patients with different liver function grades showed no significant difference between grade A and grade B. However, a considerable difference was observed between grade C and grade A or grade B. The linear equation between PVP and PVF is PVP = 1.8176 ± 0.0023 PVF. In clinical practice, it is very convenient to use color ultrasound and the linear equation to calculate PVP. With an increase in liver function grade, PVV of patients gradually slowed down, and the correlation between PVP and PVV was only significantly different in patients with grade A (*P* < 0.05). This is consistent with the results of PVV detection by Doppler ultrasound. Therefore, the dynamic monitoring of PVV is helpful for the diagnosis and evaluation of liver function in patients with cirrhosis, which has potential clinical value.

## 5 Conclusions

A linear relationship was observed between PVP and PVF or PVV, but only some PHT patients with specific liver function grades had significant differences. As a means of clinical assessment of PHT in cirrhosis, Doppler ultrasound has the advantages of noninvasiveness and efficiency. It has a good prospect in noninvasive detection of PHT in cirrhosis. In cirrhotic PHT patients, the hemodynamic parameter PVD cannot be used as an accurate means to evaluate PVP. However, PVF and PVV were correlated with PVP and had significant difference only in partial grades of cirrhotic PHT patients. Hemodynamic parameters are related to PVP to some extent, but its indicators still need to be confirmed by further or broader sample size research. Also, PVD is not a sensitive index to evaluate PVP, and the increase in PVD is not necessarily related to PHT in cirrhosis. In clinical practice, PVD is only a reference index, not an accurate method to evaluate PVP. It is concluded from this study that the measurement of these hemodynamic indexes using Doppler ultrasound may be helpful in the prognosis of liver cirrhosis and in prioritizing the allocation of liver transplantation and other treatment strategies.
